# FAIR-Compliant Database for Soil Erosion Studies: The Marganai Forest Experiment

**DOI:** 10.1038/s41597-025-04797-0

**Published:** 2025-04-02

**Authors:** Filippo Giadrossich, Ilenia Murgia, Enrico Guastini, Antonio Ganga, Simone Di Prima, Laura Chessa, Raffaella Lovreglio, Roberto Scotti

**Affiliations:** 1 Nuoro Forestry School, Dipartmento di Agraria, University of Sassari, Viale Italia, 39a, Sassari, 07100 Italy; 2 Dipartimento di Scienze e Tecnologie Agrarie, Alimentari, Ambientali e Forestali, University of Florence, Via San Bonaventura, 13, Florence, 50145 Italy; 3 Dipartimento di Architettura, Design e Urbanistica, University of Sassari, Bastioni Marco Polo, 77, Alghero, 07041 Italy; 4 Scuola di Scienze Agrarie, Forestali, Alimentari ed Ambientali, University of Basilicata, Viale dell’Ateneo Lucano, 10, Potenza, 85100 Italy; 5 Area Science Park, Loc. Padriciano, 99, Trieste, 34149 Italy

**Keywords:** Hydrology, Forestry

## Abstract

The ‘2018 Marganai Forest Soil Erosion Experiment Database’ is a comprehensive collection of measures taken during scientific experiment trials designed to investigate the effects of forest canopy coverage on soil erosion under intense artificial rainfall, four years after coppicing. The investigation involved the establishment of eight paired plots with and without forest canopy coverage, subjected to artificial rainfall simulation aimed to measure the amount of sediment transported by runoff. The work represents a valuable resource for researchers interested in understanding the complex implications of forest management practices on soil erosion. The paper, produced using Quarto in a Gitlab-based RStudio project, is an example of ‘reproducible research’ documenting that the database provides detailed information on the experimental setup as well as on the range of different measurements that have been collected. The database, produced using NFS-DataDocumentationProcedure, is stored in an SQLite file, extensively exploiting the relational properties of the engine, enhancing data accessibility, interoperability and reusability.

## Background & Summary

### Paper background

The paper presents the “Experiment of water runoff and soil erosion with and without forest canopy cover under intense artificial rainfall” database, curated and published as open data^1^. The database and the accompanying pdf document represent an application example of NFS-FAIR-DDP^2^, a “Data Documentation Procedure” aiming to promote open data FAIRness. FAIR sharing of the raw data that research produces and uses is recognized to be essential for research reproducibility^3,4^, nonetheless the process is proceeding slowly and with a limited “knowledge sharing” will, particularly in the field of forestry.

NFS-FAIR-DDP^2^ is a tool developed with the objective of facilitating the archival of research data in a verified and well documented relational structure. Data integrity, coherence, and understandability are well supported, as keys are mandatory, as is the completion of the description column in the data dictionary tables. Hence the semantics that the data bear are made truly machine accessible and reusable. The procedure requires also the completion of DataCite metadata^5^ and of other specific metadata. The database that the paper presents constitutes an example output of NFS-FAIR-DDP.

### Experiment background

Coppice forestry, which involves repeatedly cutting trees to produce new growth, has both benefits and challenges related to soil erosion risk. The dense stem networks and extensive root systems in coppice forests provide strong protection against soil erosion^6^. However, the clear-cutting process can temporarily increase erosion risk by reducing water infiltration and increasing runoff.

The Marganai forest has been extensively coppiced for several centuries until firewood was essential for the mining industry. After some decades of inactivity, the regional forest administration started implementing a forest management plan that began to coppice again on limited surfaces, activating local workforces and feeding the local demand for firewood and pellets. Due to protesters raising concerns for the potential soil erosion^7,8^, the plan was interrupted and could not be completed.

The project *Sostenibilità ambientale e socioeconomica delle utilizzazioni forestali nei cedui del Marganai* (Environmental and socio-economic sustainability of the forestry operations undertaken in Marganai coppices), has been financed by the autonomous regional administration of Sardegna through a research proposals call, opened in February 2016, leveraging on resources devoted to support the Sulcis area, including the Marganai forest, particularly impacted by the de-industrialization process. The project, presented to the call by Giadrossich F. (code: SULCIS-820965) for an overall budget of 187.500,00 EUR, has been financed for 150.000,00 EUR.

The paragraph “Context: Study Area and Forest Management from the 1850s to the Present” in^9^ details the experiment background while the paper concerns mainly the socio-economic side of the research and represents one of the main project output.

Following work presents and explains the background of the database that collects the experimental measures and observations the project produced, providing a detailed documentation of the environmental side of the research. A first version of the database was published in 2022^10^. Present version includes more detail and reflects the evolution of the NFS-FAIR-DDP project. The experimental data presented in the database focus on the related measurement of artificial rainfall intensities and consequent runoff and erosion production in pairs of plots with and without tree canopy cover. The experimental conditions are documented in detail with extensive soil analyses.

## Methods

### The study area

**Fig. 1:**
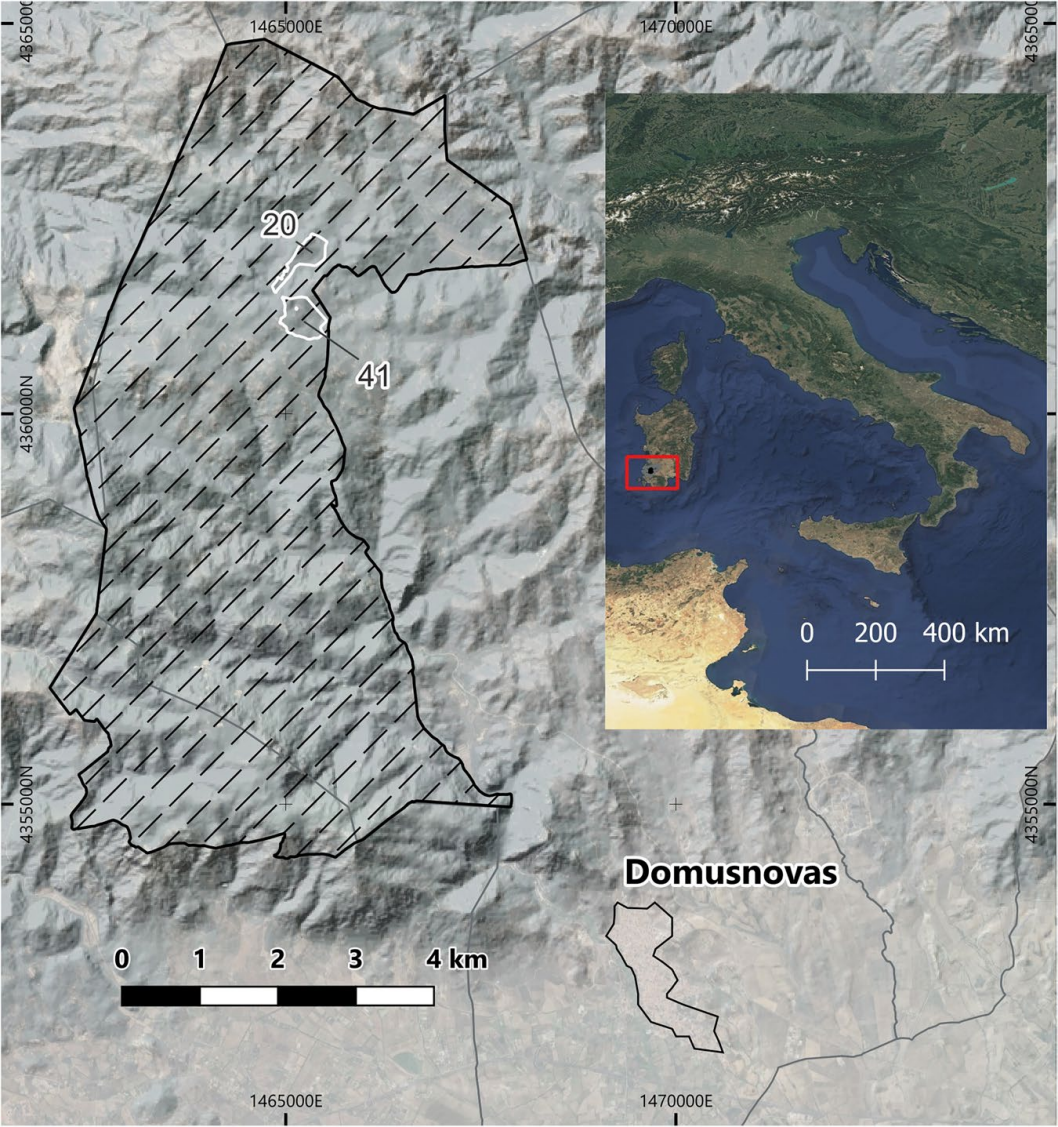
Study area geographical position. The white line represents the boundary of the forest compartments, the black line the boundary of the Marganai State Forest. Basemap: data elaboration + google satellite, © Google (2025). Geographic frame: Sentinel-2 cloudless https://s2maps.euby EOX IT Services GmbH. Licensed under CC BY 4.0 https://creativecommons.org/licenses/by/4.0/.

Experimental plots are located in the Marganai State Forest, SW Sardegna, Italy (Fig. 1). Specific toponyms and coordinates of the selected forest compartments are displayed in Table 1.

**Table 1:** Reference to the forest management plan.

(a) Relation: ‘ForestCompartments’			
ForCompId	Lat	Long	Toponym
20	39^°^24′11.9″N	8^°^35′29.9″E	S’Isteri
41	39^°^23′54.3″N	8^°^35′55.8″E	Su Caraviu

(b) Attributes			
caption	attribute	attType	unit
Compartment Id in the Marganai forest management plan (D.R.E.Am. Italia & R.D.M. Progetti, 2014)	ForCompId	id	—
Latitude	Lat	num	deg
Longitude	Long	num	deg
Forest compartment local toponym	Toponym	txt	—

In this area, the bedrock consist in limestone. Woody species are mainly represented by *Quercus ilex*, in association with *Arbutus unedo* and *Phyllirea latifolia*. Surrounding forests compartments are typical aged coppices where the time elapsed since last cut greatly exceeds the customary rotation period. Coppicing in the two considered forest compartments was conducted between 2011 and 2014, releasing approximately 150 standards per hectare. Standards are stems released during coppicing operations. During field surveys, conducted four years after the wood was cut (spring 2018), stump sprouts were observed to be 2-3 m high, with their canopies covering about 50% of the soil surface. For further details on the data, please refer to^11^.

### Experiment devices

**Fig. 2:**
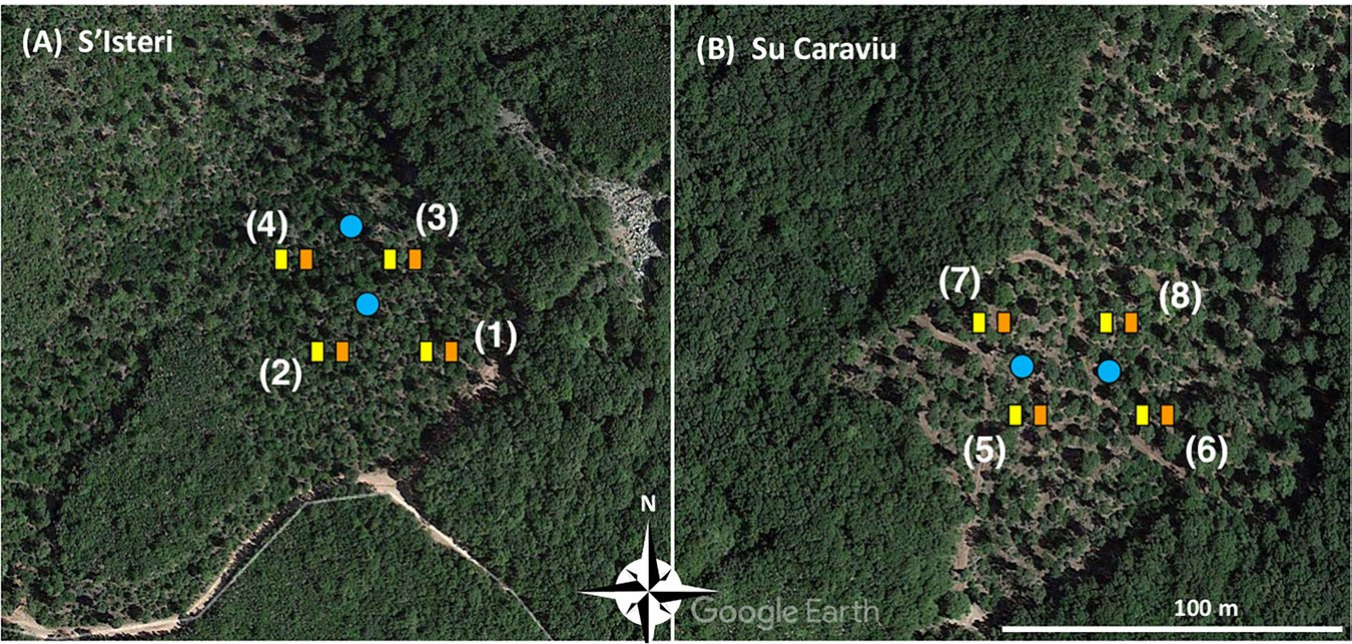
Blocks and plots position on aerial images. Numbers 1 to 8 represent blocks. Yellow and orange rectangle represent With (W) and WithOut (WO) canopy cover plots. Blue points localize the single ring water infiltration measurement spots (© Google Earth).

To deploy the experiment two sites were selected in each of the two compartments. In each site a block of two contrasting (with and without cover) plots were delimited (see Fig. 2(b-c)).

Plots, identified combining Block and Cover codes (Table 2), are listed in Table 3, while the areal extensions are computed later (see Table 4).

**Table 2:** Canopy cover characterising the plot pais in each Block.

(a) Relation ‘Cover’	
cover_id	cover
WO	without tree canopy coverage
W	with tree canopy coverage

(b) Attributes			
caption	attribute	attType	unit
canopy cover label	cover_id	id	—
canopy cover presence description	cover	txt	—

**Table 3:** Plots details.

(a) Relation:‘Plots’		
Block	cover_id	Slope_pct
1	WO	29
1	W	29
2	WO	33
2	W	40
3	WO	36
3	W	27
4	WO	40
4	W	44
5	WO	33
5	W	38
6	WO	44
6	W	58
7	WO	58
7	W	64
8	WO	51
8	W	49

(b) Attributes			
caption	attribute	attType	unit
Experiment replication Id	Block	id	—
Canopy cover label	cover_id	id	—
Local slope gradient	Slope_pct	num	%

**Table 4:** Reference data for the rain measurement devices.

(a) Relation: ‘RainGaugeReferenceArea’ (3 not shown rows!)		
RainGaugePos	RepresentedArea	Caption
U	3750	central, uphill
R	5625	right, midway
L	5625	left, midway
E	0	external

(b) Attributes			
caption	attribute	attType	unit
Id of rain gauge position	RainGaugePos	id	—
Portion of the plot that the rain gauge represents	RepresentedArea	num	mm^2^
Description of rain gauge position with respect to the plot and the slope	Caption	txt	—

**Fig. 3:**
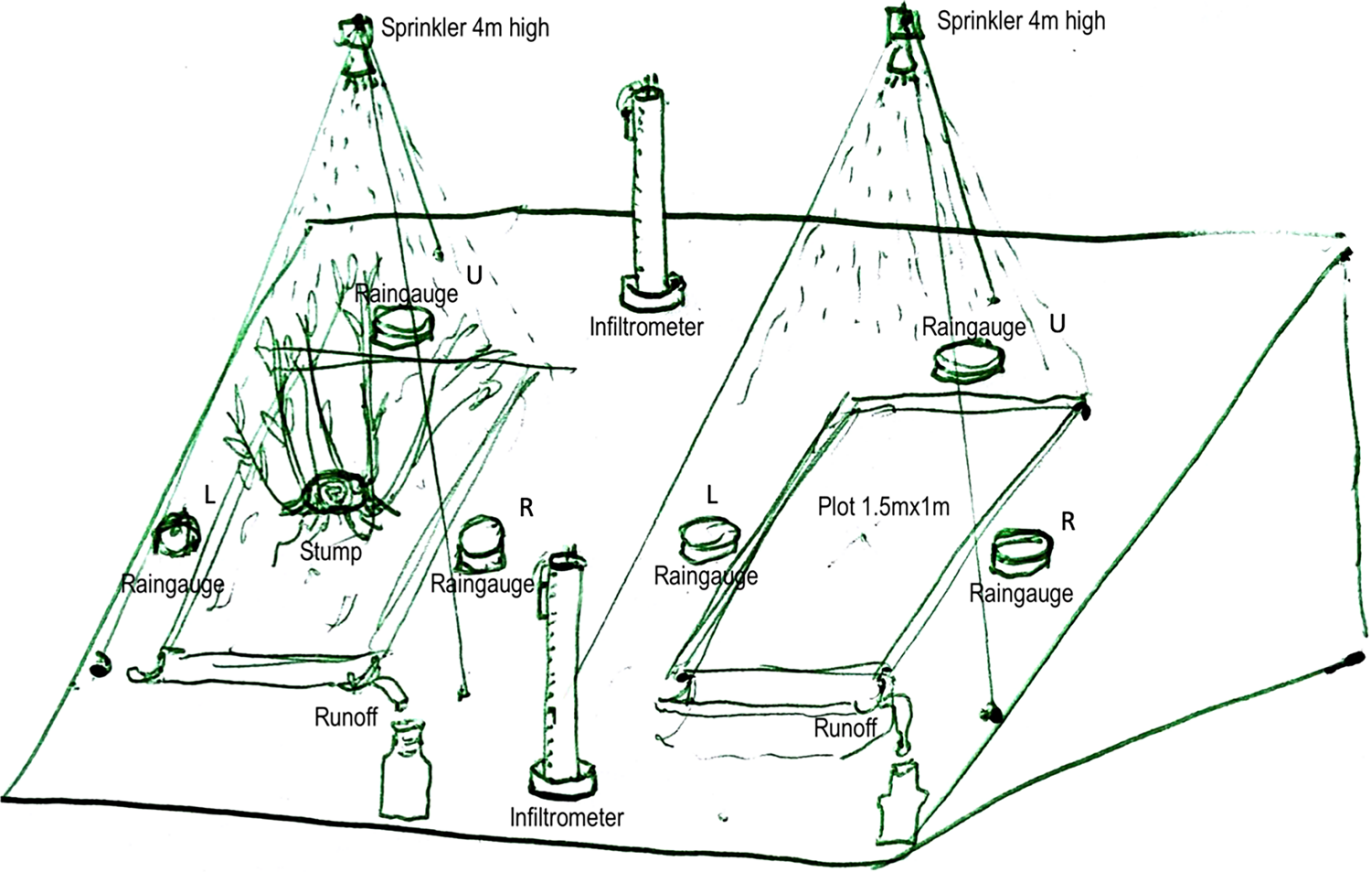
Blocks and plots installation schema (L/U/R are, respectively, left/upper/right raingauges. Drawn by Filippo Giadrossich, 2018. Licensed under CC BY 4.0 https://creativecommons.org/licenses/by/4.0/.

The experimental device, drafted in (Fig. 3), consists of couples of plots, located in a close neighbourhood. The one with cover (W) had a stump in the centre, the other was without cover (WO). Over each plot, with the help of a wooden tripod having a modular structure similar to the one used in^12^, a nozzle was positioned, centrally with respect to the plot surface, 4 m above the ground. Authors^13^ and^14^ have experimentally evaluated that a nozzle of this type, at 4 m above the ground, produces drops with characteristics that resemble well those of natural rain drops.

The nozzle used was a calibrated Lechler nozzle (mod. 490.888) with a spray angle of 120 degrees and a water pressure of 1.5 bar. The uniformity of the rainfall distribution was ensured by preliminary laboratory tests, measuring the rainfall at different points within the nozzle’s range of action. However, during the experimental rain simulations, three rain gauges were positioned, one at the top and two on the sides of each plot. To correctly weight the contribution of each rain gauge, the “RepresentedArea” is specified in Table 4.

**Fig. 4:**
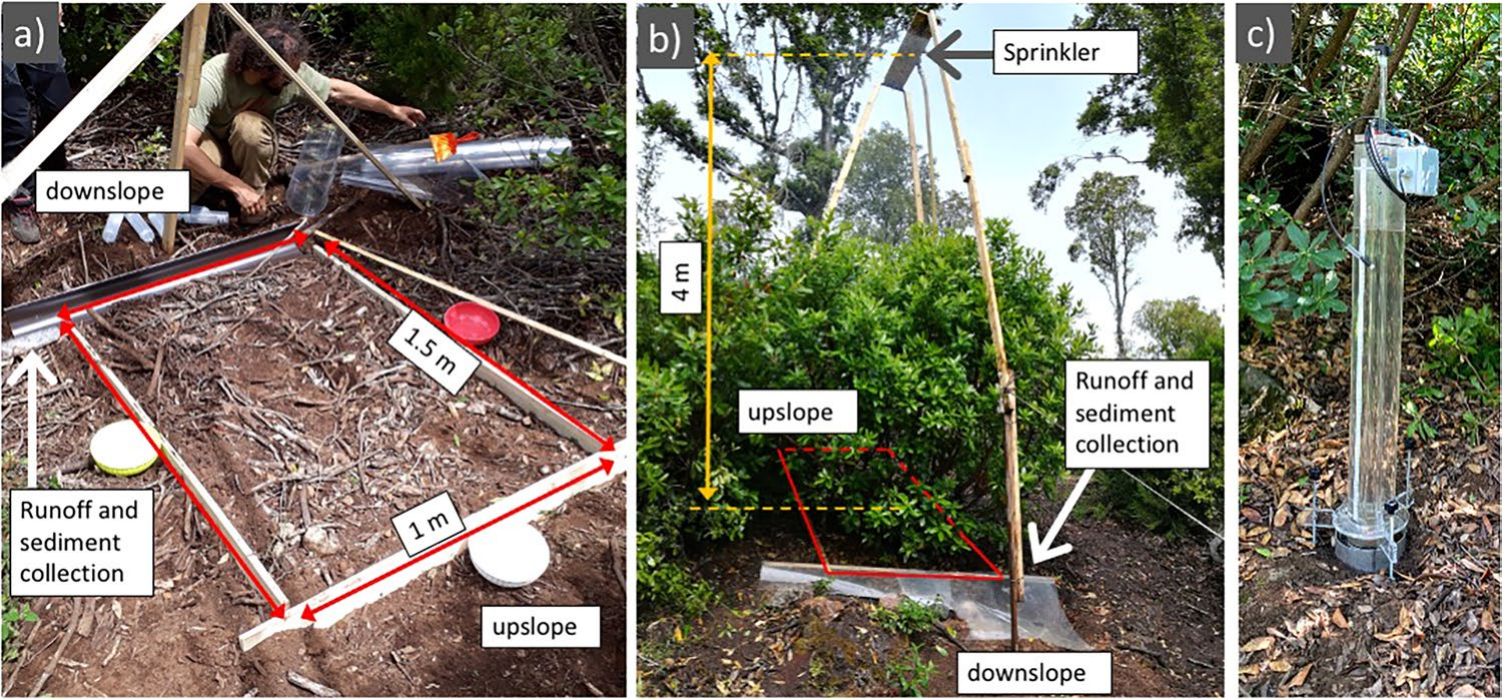
Blocks and plots installation details. Rainfall simulation setup: (**a**) plot without (WO) sprouts crown cover, (**b**) plot with (W) crown cover, (**c**) Automated single ring infiltrometer for Beerkan tests.

The plot soil was fenced on all sides to a depth of about 15 cm. Downhill, a water collector was placed to collect all surface drainage and erosion caused by (artificial) rain. To provide a detailed characterization of the hydraulic status and behaviour of the soils different infiltrometric measures were taken just beside the plots: accurate cylinder-based measures as well as simpler scoop-based measures of soil water content. Photos in Fig. 4 document some moments of the deployment of the experimental device on a plot.

### Experimental design

The experiment consists in a sequence of trials replicating, for each plot, the artificial rainfall generation following the protocol detailed in Table 5.

**Table 5:** Experimental setup.

(a) Relation: ‘Trials’			
Trial	RainStartTime	RainStopTime	Comment
0	0	0	Measurement before experimental raining started
1	0	30	First continuous artificial rain period
2	45	75	Second continuous artificial rain period
3	90	120	Eventual additional continuous artificial rain period

(b) Attributes			
Caption	Attribute	attType	Unit
Codes commented in Trials table	Trial	id	—
Trial start time	RainStartTime	num	min
Trial stop time (15 min with no rain separates successive trials)	RainStopTime	num	min
Trial time comment	Comment	txt	—

**Fig. 5:**
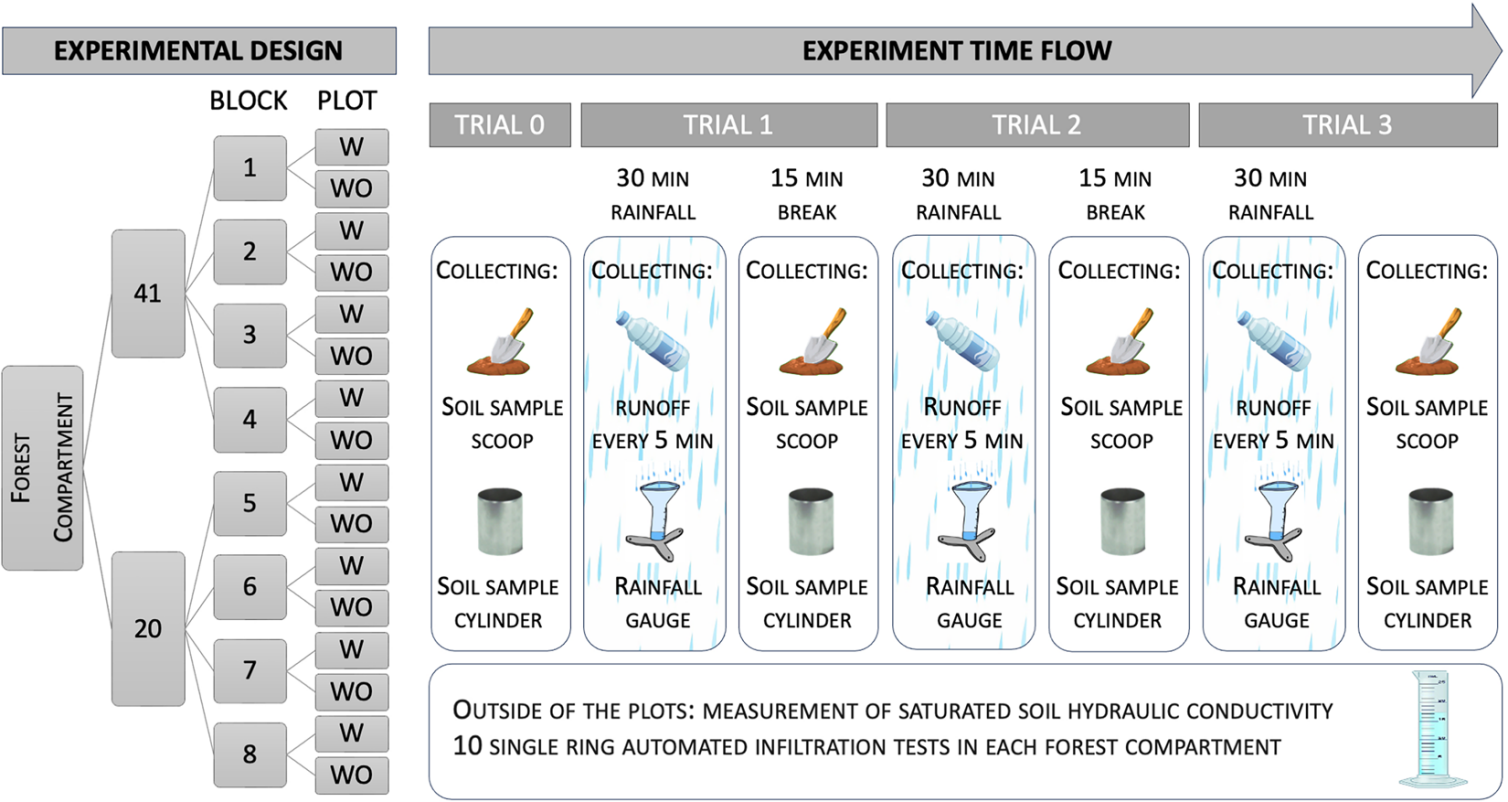
Graphical synthesis of the experiment.

In each plot, a trial consists of 30 min of continuous rainfall. Between trials, 15 min of no rain were observed. Trial 0 was only to evaluate the soil water content before the start of the artificial rain. Trials 1 and 2 were carried out on all plots simulating heavy rainfall that occurs in relatively dry soil (Trial 1) and on wet soil (Trial 2). Trial 3 represents an additional effort focused on the critical case plots not protected by tree canopy (WO, without cover plots). The scheme in Fig. 5 shows a graphical synthesis of the experimental design.

### Experiment implementation

#### Analysis of soil characteristics

Soil characterisation is essential for assessing the impact of rain. Soil texture, bulk density, and saturated hydraulic conductivity are interrelated parameters that significantly influence the processes by which rainfall is converted into surface runoff and the related occurrence of soil erosion.

Soil samples were collected before artificial rainfall (Trial = 0) and at the the end of all trials (1, 2, eventually 3) (Figure 5). The protocol included different sampling procedures and analysis. The soil bulk density was obtained using volumetric cylinder method^15^ by collecting soil samples in a 100 cm steel cylinder, first measuring the mass of the wet soil and then drying the samples in an oven at 105 degrees Celsius for two days. To increase the number of gravimetric water content samples, following method described in^16^, also scoop-based samples were collected. The main parameters characterizing the different procedures adopted are included in Table 6.

**Table 6:** General sampling data and soil sampling parameters.

(a) Relation:‘Sampling_param’		
Param	MeasurementUnit	Value
plot_level_width	m	1
plot_length	m	1.5
soil_sample_cylinder_volume	cm^3^	100
texture_SubSample	g	20
skeleton_min	mm	2
coarse_sand_min	mm	0.2
fine_sand_min	mm	0.02
silt_min	mm	0.002
clay_max	mm	0.002
Collection date for soil samples connected to hydraulic conductivity test	date	2020-09-16
Drying date of soil samples connected to hydraulic conductivity test	date	2020-10-08

(b) Attributes			
Caption	Attribute	attType	Unit
Parameter name	Param	txt	—
Measurement unit used for parameter value	MeasurementUnit	txt	—
Parameter value	Value	txt	—

**Fig. 6:**
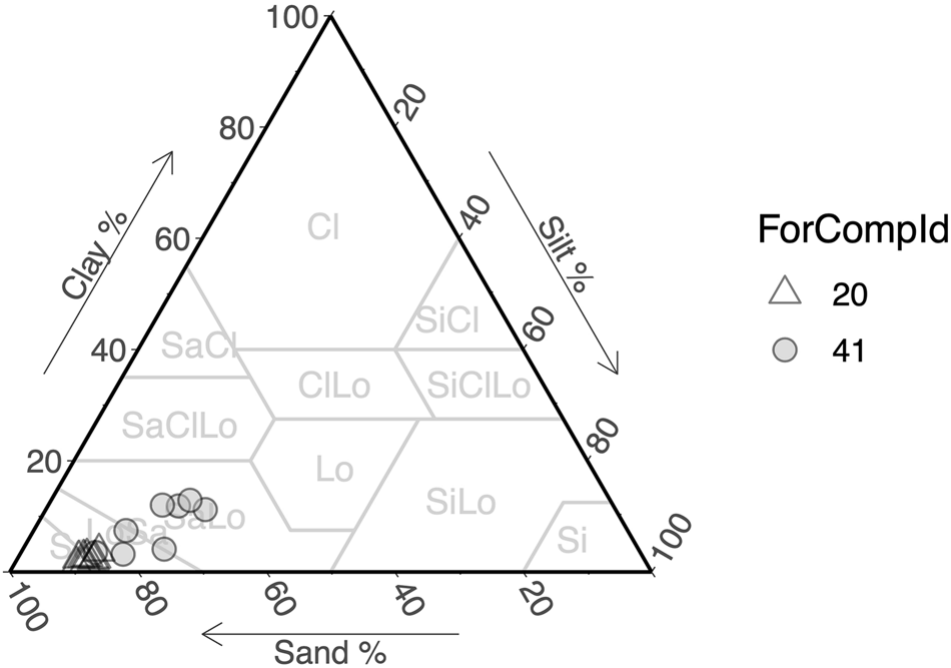
Position of the samples within the USDA (2017) soil texture classification system, by “forest compartment”.

Soil texture was evaluated, pooling trial samples within each plot, with reference to the ISO 11277:2020 soil classification system (Table 6), following the standard methodologies. Using sieve, sedimentation and pipette method and incorporating sodium hexametaphosphate as a dispersing agent to ensure complete disaggregation of soil particles. Sand was separated by wet sieving, dried, and weighed. The finer silt and clay fractions were analyzed using aliquots extracted at specific sedimentation intervals based on Stokes’ law, dried, and weighed. The two forest compartments are characterised by sandy soils with slight differences between them (Fig. 6, Table 7).

**Table 7:** Pooled scoop-based samples.

(a) Relation: SoilSampling_texture (14 not shown rows!)							
Block	cover_id	Skeleton_tot	OrganicM_ss	CoarseSand_ss	FineSand_ss	Silt_ss	Clay_ss
1	WO	683.95	2.90	5.08	6.51	3.48	2.03
1	W	436.48	3.88	6.19	6.41	2.32	1.20

(b) Attributes			
Caption	Attribute	attType	Unit
Experiment replication Id	Block	id	—
Canopy cover label	cover_id	id	—
Weight of the skeleton fraction of total pooled scoop-based soil samples	Skeleton_tot	num	g
Organic fraction of the pooled scoop-based soil subsample	OrganicM_ss	num	%
Weight of the coarse sand fraction of the pooled scoop-based soil subsample	CoarseSand_ss	num	g
Weight of the fine sand fraction of the pooled scoop-based soil subsample	FineSand_ss	num	g
Weight of the fine silt fraction of the pooled scoop-based soil subsample	Silt_ss	num	g
Weight of the clay fraction of the pooled scoop-based soil subsample	Clay_ss	num	g

#### Soil saturated hydraulic conductivity

The soil saturated hydraulic conductivity (K_s_) was evaluated with 20 tests, 10 for each forest compartment, measuring the rate at which water moves through the soil under saturated conditions, utilizing the single-ring automated field test method as described by^17^. During the estimation most of the cumulative infiltration curves showed evidence of soil water repellency (SWR), with attenuation of infiltration rates at early times and a convex shape with an increasing slope with time. To account for SWR we used an adapted Beerkan Estimation of Soil Transfer parameters (BEST) method, called BEST-WR. This method, proposed by^18^, uses an empirical exponential scaling factor that describes the rate of attenuation of infiltration rate due to SWR^19^. Calculations were performed using the R script available at https://bestsoilhydro.net. Due to the log-normal distribution of the K_s_ values, geometric means were computed^20^ and, following^21^, the appropriate CV expression for a log-normal distribution was employed. The Table 8 presents a synthesis of the measures. The difference between the forest compartments is not relevant, it reflects the small differences in the soil textures and is irrelevant with respect to effects of the experimental rainfall.

**Table 8:** Synthesis of single ring hydraulic conductivity tests.

(a) Relation:‘Soil_SatHydrConduct’					
ForCompId	NumObs	Ks_min	Ks_max	Ks_geometricMean	Ks_CV
20	10	222.2	972.5	401.5	42.8
41	10	186.9	1436.9	487.4	58.9

(b) Attributes			
caption	attribute	attType	unit
Compartment Id in the Marganai forest management plan (D.R.E.Am. Italia & R.D.M. Progetti, 2014)	ForCompId	id	—
Number of hydraulic conductivity tests	NumObs	num	—
Hydraulic conductivity min value	Ks_min	num	mm*h^−1^
Hydraulic conductivity max value	Ks_max	num	mm*h^−1^
Hydraulic conductivity geometric mean (skewed ditributions)	Ks_geometricMean	num	mm*h^−1^
Coefficient of variation of hydraulic conductivity measures	Ks_CV	num	%

#### Monitoring rainfall

The rain intensities (Table 9) were measured for each trial in each plot by the three rain gauges positioned just outside of plots boundaries (Figure 3). Plot level intensities were estimated as averages weighted by the area each rain gauge represents (Table 4). Additional rain gauges were placed about 3 m away from the plots, avoiding artificial rain, to monitor the contribution of occasional natural rainfall. As documented in the database (in Table 9 and Table 4, where RainGaugePos = E means external, additional rain gauge), the natural rain contribution has been recorded and ultimately appears to be irrelevant. Other causes of variability of the artificial rain intensity like pump pressure variations and wind, were considered and had no reportable effect. The pump pressure gauges were carefully monitored by the operator during each trial run. During some of the trials, the wind slightly disturbed the artificial rainfall, but with effects considered to be negligible.

**Table 9:** Collected rainfall measurements.

(a) Relation:‘RainGauge’(118 not shown rows!)				
Block	cover_id	Trial	RainGaugePos	RainIntensity
1	WO	1	R	37.25
1	WO	1	L	38.28
1	WO	1	U	49.91
1	WO	2	R	30.69

(b) Attributes			
caption	attribute	attType	unit
Experiment replication Id	Block	id	—
Canopy cover label	cover_id	id	—
Codes commented in Trials table	Trial	id	—
Id of rain gauge position	RainGaugePos	id	—
Rainfall measured in each rain gauge	RainIntensity	num	mm*h^−1^

#### Monitoring runoff and soil erosion

**Fig. 7:**
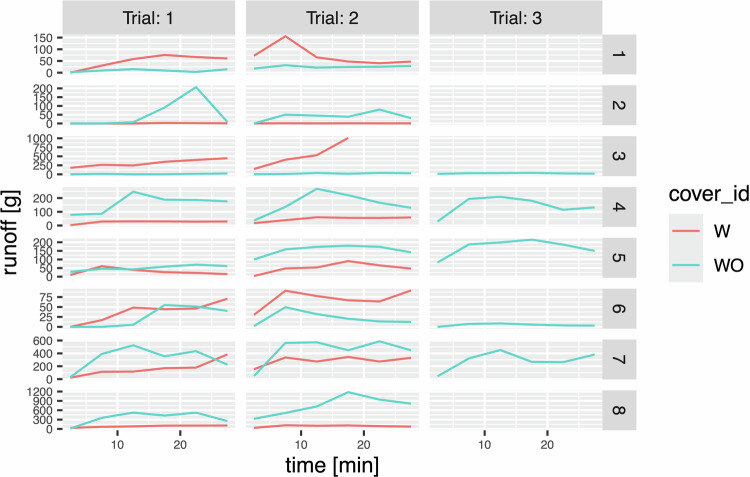
Runoff collected, every 5 min, during the trials in each plot of each block.

Runoff and sediment were manually collected every 5 min in 0.5 l bottles, on the lower side of the plot, using one bottle for each 5 min interval (Table 10, Fig. 7).

**Table 10:** Surface water runoff.

(a) Relation:‘Runoff’(216 not shown rows!)					
Block	cover_id	Trial	StartTime	EndTime	Runoff
1	W	1	0	5	0.10
1	W	1	5	10	30.27
1	W	1	10	15	58.15
1	W	1	15	20	75.58
1	W	1	20	25	66.73
1	W	1	25	30	60.62

(b) Attributes			
caption	attribute	attType	unit
Experiment replication Id	Block	id	—
Canopy cover label	cover_id	id	—
Codes commented in Trials table	Trial	id	—
Start time of runoff water collection trial subperiod	StartTime	num	time
End time of runoff water collection trial subperiod	EndTime	num	time
Weight of runoff water collected in the trial subperiod	Runoff	num	g

In one case, a bottle filled completely in less than 5 min, necessitating the use of a second bottle, but since the bottles were precisely numbered in advance and this event was unexpected, no data were recorded during the last minutes of this test (Test 2, Block 3). Due to the general scarcity of runoff and especially of sediment the values of the individual samples were too small to measure. Sediment quantification had to be performed by aggregating the sediment collected over entire 30 min trials to obtain a measurable amount, pooling and drying the 5 min runoff collections for each trial in each plot (Table 11). In the collected solid matter the organic fraction was separated from the sediment following Walkley-Black method^22^.

**Table 11:** Solid matter in the water flow.

(a) Relation:‘Sediment’(31 not shown rows!)				
Block	cover_id	Trial	Sediment	OrganicMatter
1	WO	1	0.6908	15.1271
1	WO	2	0.8580	24.9998
1	W	1	1.0840	22.5675
1	W	2	1.4960	28.2638
2	WO	1	0.3240	19.9493
2	WO	2	1.7790	25.9373

(b) Attributes			
caption	attribute	attType	unit
Experiment replication Id	Block	id	—
canopy cover label	cover_id	id	—
Codes commented in Trials table	Trial	id	—
Sediment contained in the runoff water collected in the trial	Sediment	num	g
Organic fraction in the runoff water collected in the trial	OrganicMatter	num	%

## Data Records

The database is available at PANGAEA data repository^1^ that distributes georeferenced data from earth and environmental sciences, focusing on observational and experimental data^23^. The publication includes two records: the SQLite database file and a “pdf” documentation file. Both are produced using the NFS-FAIR-DDP. The documentation provides an overview of the structure and detailed contents of the database. SQLite’s widely used, open format combines ease of access to data with the ability to ensure internal consistency of the data tables. The procedure used to produce the files further extends this advantages complementing the basic data with rich metadata following the standard DataCite schema^24^.

## Technical Validation

Fieldwork often presents challenges beyond our control, but we made every effort to ensure that the data we collected was reliable and consistent. The validation of data relied on strict adherence to established procedures, continuous supervision, and meticulous attention to the accuracy and precision of sampling methods. This involved careful timing and sequencing of artificial rainfall trials, as well as the precise location of sampling points within the experimental plots.

The placement of plots was thoughtfully chosen to reflect the site’s representative characteristics while ensuring that the soil surface remained undisturbed. Consequently, the litter, sticks, and canopy cover observed during the trials were naturally developed or deposited since the most recent coppice cutting, preserving the authenticity of the site’s natural conditions.

Soil samples were collected with great care, strictly adhering to the protocol outlined in the Methods section. To ensure the accuracy of the artificial rainfall, we used an internally calibrated Lechler nozzle (mod. 490.888), which had undergone laboratory testing to confirm its ability to deliver spatially uniform rainfall. Water for the experiments was supplied by medium-sized tankers equipped with pumps to maintain a steady flow. Pressure was monitored using gauges placed at strategic points, including the tanker truck’s exit, the base of the wooden tripod, and near the nozzle, ensuring consistent water pressure throughout the trials.

During field measurements, runoff and sediment were carefully collected in bottles, which were weighed using precision scales. Scoop, soil sample cylinders, and rainfall gauges were also employed to gather data. Once collected, samples were securely sealed and transported to the laboratory, where standard analysis procedures were applied. These analyses included measurements of bulk density, soil texture, runoff, sediment composition, and organic carbon content in the sediment, all conducted in accordance with recognized methodologies^15,16^. Data from single ring infiltrometer tests^18^ were also processed and analyzed to provide further insights into soil infiltration rates.

Beyond field and laboratory work, our efforts focused on ensuring data integrity and compliance with FAIR principles through the use of the NFS-FAIR-DDP. During the data entry process into the spreadsheet template, values were meticulously double-checked to verify accuracy. The NFS-data-documentation procedure was then employed to validate table relationships, ensuring the correct implementation of primary and foreign keys, which was critical for maintaining the consistency and reliability of the database structure. The procedure also included a thorough review of metadata to ensure adherence to standardized formats and completeness. By transforming the dataset into a rigorously referenced SQLite database, the NFS-DDP established a robust framework that safeguards data quality while enhancing its findability, accessibility, interoperability, and reusability^25^. These efforts represent our commitment to transparency, reliability, and the effective sharing of research data.

## Usage Notes

**Fig. 8:**
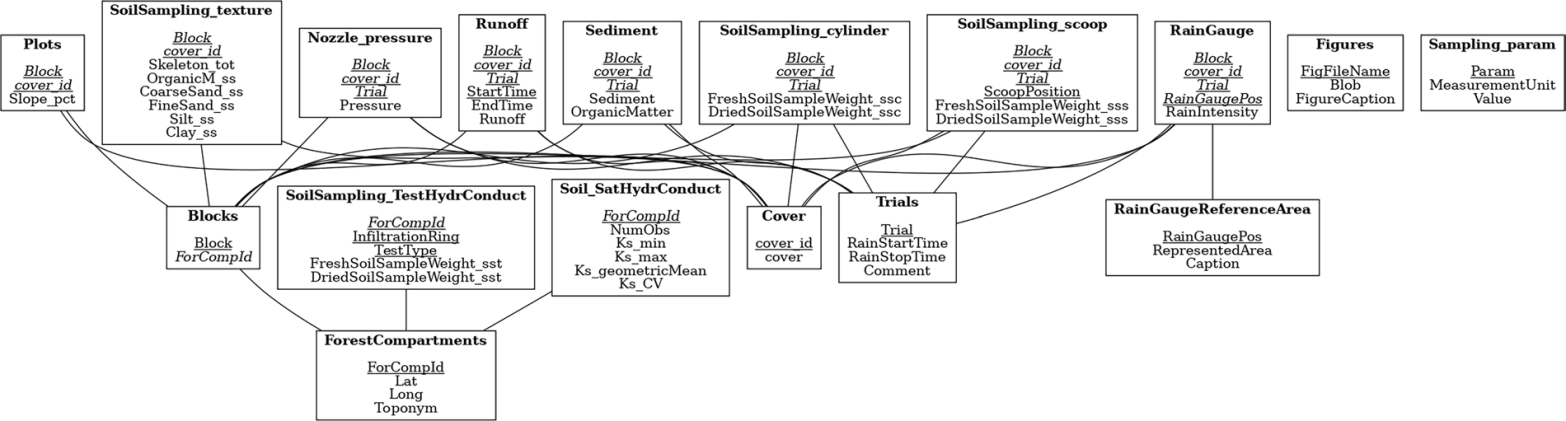
Database schema as Entity-Relationship Diagram.

The database and the accompanying documentation offer detailed standard guidance for the use of the data. Both include the “ERD-like” schema representation (Entity-Relationship Diagram, Fig. 8). This tool presents a map of all the data and the connections among tables, providing a detailed structural understanding of the set.

Present paper has been produced using Quarto^26^ and R^27^, in Rstudio^28^. In this environment, SQLite files are typically accessed via RSQLIite and DBI packages. The function dbMakeTibble(table_name, conn){=}, in Algorithm 1, is a wrapper around the basic dbReadTable(conn, name){=} function. It demonstrates how to access the data, controlling that the request is valid and avoiding the creation of unwanted files. Algorithm 1 shows the code that reads the “Sampling param” table values (Table 6) from the database to make them available for calculations.

### Algorithm 1

Extracting parameter values and creating corresponding variables. 

The code lines in Algorithm 2 provide a further example, extracting the ERD image (Fig. 8).

### Algorithm 2

Example code chunk extracting the ERD image from the DB. 

Code in Algorithm 3 demonstrates how to extract from the database the documentation, included among the metadata, to present the “title" and the “experiment synthesis”.

### Algorithm 3

Extracting and presenting example metadata.

## Data Availability

Quarto is a popular tool for reproducible research^26^. A Quarto file is a markdown document containing text and code chunks. Software code used to produce the present parer is hence embedded in the Quarto document that generated it. The Quarto file and the accompanying software used for the production of this paper are accessible in the Gitlab repo (https://gitlab.com/NuoroForestrySchool/nfs-data_documentation_procedure-examples/2018-marganai-forest-soil-erosion-experiment-database/-/tree/Scientific_data). The repository offers a complete guide to the exploitation of the database.
